# Comments on: “Comparison of Methods Study between a Photonic Crystal Biosensor and Certified ELISA to Measure Biomarkers of Iron Deficiency in Chronic Kidney Disease Patients”

**DOI:** 10.3390/s20041126

**Published:** 2020-02-19

**Authors:** Naseem Abbas, Rizwan Ali Naqvi

**Affiliations:** 1 Department of Mechanical Engineering, Chung-Ang University, 84 Heukseok-ro, Dongjak-gu, Seoul 06974, Korea; 2 Department of Unmanned Vehicle Engineering, Sejong University, Seoul 05006, Korea

We recently read, with great interest, the article entitled “Comparison of Methods Study between a Photonic Crystal Biosensor and Certified ELISA to Measure Biomarkers of Iron Deficiency in Chronic Kidney Disease Patients” [1]. After reading this article, we would like to address some issues.

The authors reported the total analytical error of a photonic crystal (PC) biosensor in the determination of ferritin and soluble transferrin receptor (sTfR) as biomarkers of iron deficiency anemia in chronic kidney disease (CKD) patients against certified ELISAs [1]. They calculated the inherent imprecision using Equation (1):

σ^2^(δ) = σ^2^_T_+σ^2^_R_ (1)

where σ^2^_T_ is the variance of the test method (i.e., PC) and σ^2^_R_ is the variance of the reference method (i.e., ELISA) for each biomarker, and σ^2^(δ) is the total inherent imprecision of the test and reference methods together [2]. Difference plots were constructed to determine whether the PC biosensor measuring each biomarker, was statistically differently or not from the ELISA method. In this test, the null hypothesis was that the measured differences for all samples are zero and the constant analytical standard deviation are presumed to be equal to σ_T_ + σ_R_. Therefore, when the two methods are identical, it is expected that 68% of differences are distributed around 0 ± 1σ(δ), and 95% of differences are distributed between 0 ± 2σ(δ), as illustrated in the different plots [2]. In Figure 2, the authors draw the difference plot comparing serum ferritin and sTfR concentrations from hemodialysis patients using the PC biosensor against the certified ELISAs by claiming that inherent imprecision indicates the range in which the mean differences must fall in order to fail to reject the null hypothesis that there are no differences between methods. That is, 68% and 95% of the differences must fall between 0 ± 24 and 0 ± 48 ng/mL, Thus, in the case of ferritin, the PC assay’s inherent analytical imprecision was different than that of sTfR concentrations, respectively. The actual distribution of mean differences was 43% at 0 ± 1σ(δ) and 68% at 0 ± 2σ(δ). At the same time, the same trend was evaluated for sTfR biomarker, providing information about the actual distribution. The actual distribution for sTfR was 62% at 0 ± 1σ(δ) and 96% at 0 ± 2σ(δ). 

In their concluding remarks, by using different chronic kidney disease patients’ data, the authors claim that the PC assay’s inherent analytical imprecision was different than that of the ELISA in the case of the ferritin biomarker. However, in the case of the sTfR biomarker, there was no statistical difference between the methods. Figure 2a for ferritin and Figure 2b for sTfR indicate the difference plots comparing serum ferritin and sTfR concentrations from hemodialysis patients using the PC biosensor against the certified ELISAs. 

The problem is, Figure 2a,b indicates that for both serum ferritin and sTfR concentration cases, there is no statistical difference between the PC biosensor against the certified ELISAs. It shows a contradictory statement, as the authors claim in their published article [1] that there is a statistical difference between the reference method and the test method for ferritin, but there is no statistical difference for sTfR. For this purpose, to correct Figure 2a, we re-produce the inherent imprecision graph for serum ferritin concentration using the same data that the authors explain in their published article. “Using Equation (1), the mean bias of measuring serum ferritin with the PC biosensor was 7 ng/ml and the inherent imprecision σ(δ) of both diagnostic methods was 24 ng/mL”. Figure 2a of the article [1] should be replaced with the new Figure (Figure 2), as Figure 2a creates misleading informations for the readers of the *Sensors* journal. Figure 2 indicates clearly that “the actual distribution of mean differences was 43% at 0 ± 1σ(δ) and 68% at 0 ± 2σ(δ)”.

In this comment, Figure 2a of the published article [1] is re-numbered as Figure 1 and the re-produced Figure is denoted as Figure 2.

## Figures and Tables

**Figure 1:**
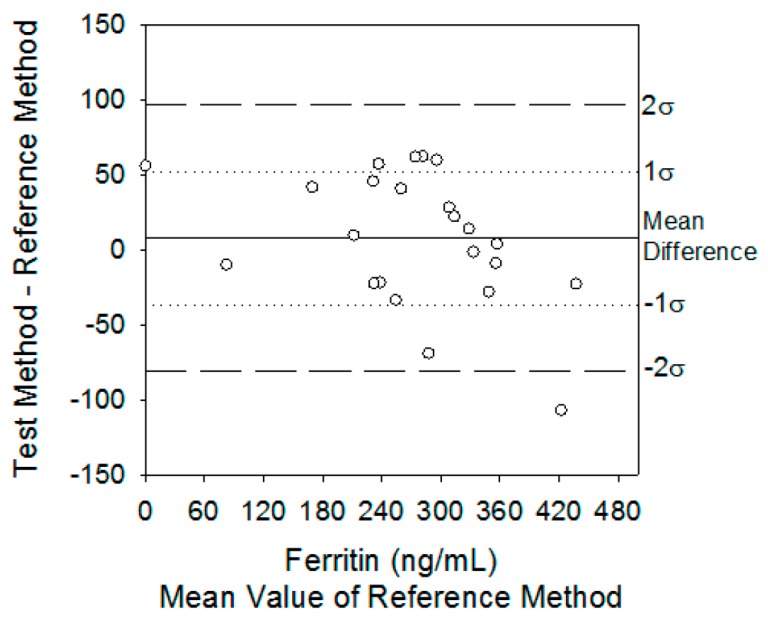
Difference plots comparing serum ferritin concentrations from hemodialysis patients using the photonic crystal (PC) biosensor against the certified ELISAs [1].

**Figure 2:**
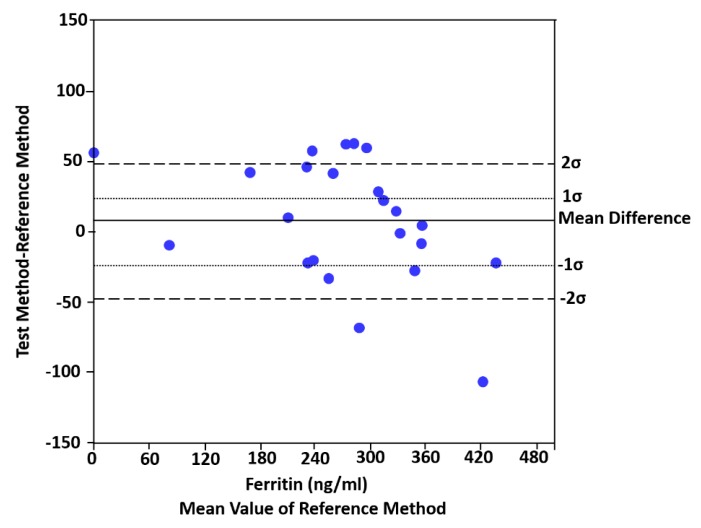
Difference plots comparing serum ferritin concentrations from hemodialysis patients using the PC biosensor against the certified ELISAs.
